# Valorization of *Prunus* Seed Oils: Fatty Acids Composition and Oxidative Stability

**DOI:** 10.3390/molecules28207045

**Published:** 2023-10-12

**Authors:** Sandra Rodríguez-Blázquez, Esther Gómez-Mejía, Noelia Rosales-Conrado, María Eugenia León-González, Beatriz García-Sánchez, Ruben Miranda

**Affiliations:** 1 Department of Analytical Chemistry, Faculty of Chemistry, Complutense University of Madrid, Complutense Avenue, 28040 Madrid, Spain; sandro08@ucm.es (S.R.-B.); egomez03@ucm.es (E.G.-M.); nrosales@ucm.es (N.R.-C.); 2 Department of Chemical Engineering and Materials, Faculty of Chemistry, Complutense University of Madrid, Complutense Avenue, 28040 Madrid, Spain; beatriga@ucm.es (B.G.-S.); rmiranda@ucm.es (R.M.)

**Keywords:** *Prunus* seed oils, peach seeds, apricot seeds, plum seeds, cherry seeds, fatty acids, antioxidant activity, oxidative stability, by-product valorization

## Abstract

*Prunus* fruit seeds are one of the main types of agri-food waste generated worldwide during the processing of fruits to produce jams, juices and preserves. To valorize this by-product, the aim of this work was the nutritional analysis of peach, apricot, plum and cherry seeds using the official AOAC methods, together with the extraction and characterization of the lipid profile of seed oils using GC-FID, as well as the measurement of the antioxidant activity and oxidative stability using the 2,2-diphenyl-1-picrylhydrazyl (DPPH) free-radical scavenging method. Chemometric tools were required for data evaluation and the obtained results indicated that the main component of seeds were oils (30–38%, w). All seed oils were rich in oleic (C18:1n9c) and linoleic (C18:2n6c) acids and presented heart-healthy lipid indexes. Oil antioxidant activity was estimated in the range IC_50_ = 20–35 mg·mL^−1^, and high oxidative stability was observed for all evaluated oils during 1–22 storage days, with the plum seed oil being the most antioxidant and stable over time. Oxidative stability was also positively correlated with oleic acid content and negatively correlated with linoleic acid content. Therefore, this research showed that the four *Prunus* seed oils present interesting healthy characteristics for their use and potential application in the cosmetic and nutraceutical industries.

## 1. Introduction

Currently, there is a high level of interest in the search for new alternatives for the recovery of waste from the agri-food industry [1,2]. During the industrial processing of fruits, a large amount of waste and by-products such as peels, stones and seeds are generated [1,3,4]. The deposition of these materials in landfill sites has significant negative food-security, economic and environmental impacts [5]. Therefore, to reduce environmental pollution along with economic losses, different research studies have reported that agri-food waste material could be exploited as a source of high-value-added compounds that could be applied in the cosmetic, pharmaceutical and nutraceutical sectors [2,3,6].

The processing of some members of the Rosaceae family in the genus *Prunus*, including peach (*Prunus persica*), apricot (*Prunus armeniaca*), plum (*Prunus domestica*) and sweet cherry (*Prunus avium*), is of great importance worldwide [7]. According to the 2023 campaign for stone fruits [8], 110,692 tons of apricot, 805,368 tons of peach, 164,685 tons of plum and 140,166 tons of cherry were produced in Spain.

All *Prunus* species are highly appreciated by consumers and, thus, they are being studied not only because of their taste, color and sweetness, but also for their nutritional composition and bioactive properties [9,10]. Their entire stone is divided into a central softer part known as the kernel or seed, and an outer hard part named the shell. Depending on the fruit, the seed of the stone accounts for approximately 15–30 wt.% of the entire stone, while the remaining 70–85 wt.% corresponds to the stone’s shell [11]. *Prunus* seeds are one of the main components discarded during the production of juices and jams, and in other processing industries. This waste constitutes a huge nutritional loss, since seeds are a good source of unsaturated fatty acids, proteins, dietary fibers, carbohydrates, polyphenols, vitamins and other bioactive compounds [12,13]. The presence of proteins, carbohydrates and dietary fibers is essential for the maintenance of human tissues, the production of neurotransmitters, the generation of energy reserves and the proper functioning of the human intestine, among others [14].

Among the main components of seeds, due to their multiple bioactivities, are the oils [12]. The levels of bioactive compounds in seed oil depends on the extraction method used. Although non-traditional techniques are currently used that reduce the use of solvents, such as cold pressing, supercritical methods, ultrasonic-assisted extraction and microwave-assisted extraction [15], the reference method par excellence is Soxhlet extraction. Due to its simplicity and high extraction yield, it is one of the most widely used techniques for the recovery of fatty acids from oils [16]. The most common solvent used in this extraction technique is *n*-hexane due to its high solubility with oils, low cost, high volatility, low boiling point and easy removal from solids [17].

*Prunus* seed oils are mainly characterized by triacylglycerides of different chemical natures. The main fatty acids found in *Prunus* seed oils are oleic (52–66%), linoleic (25–35%) and palmitic acids (3–10%) [9,18]. The high content of unsaturated fatty acids (UFA) in the oils reduces the concentration of low-density lipoproteins (LDL) which, when circulating in the body, are deposited in the blood vessels, reducing dysrhythmias, mortality caused by coronary disease and the rate of atherosclerosis, as well as blood pressure [19,20]. In addition, the high content of polyunsaturated fatty acids (PUFA) in seed oils has been related to the development and/or regeneration of cell membranes and a protective effect against dementia and Alzheimer’s disease [19,21]. Furthermore, oils are characterized by a low content of saturated fatty acids (SFA), which increase LDL protein levels and the risk of coronary heart disease [22]. A PUFA/SFA ratio below 0.45 in the diet is a risk factor for increased blood cholesterol levels [23]. 

The health quality of lipids, based on fatty acid composition, is determined using indexes. The desirable fatty acid (DFA) index provides information on the hypocholesterolemic properties (reduction of total cholesterol) of the lipids analyzed [24]. The hypocholesterolemic/hypercholesterolemic (HH) ratio can become an indicator of the cholesterol effect of a fat source. Atherogenicity (AI) and thrombogenicity (TI) indexes are also used to indicate the risk of developing cardiovascular irregularities [25]. The n-6/n-3 PUFA ratio is another important parameter to consider when establishing the nutritional value of oils as it is related to lipid metabolism, neurogenesis and cell apoptosis [12,26,27]. 

In addition to the importance of knowing the content of fatty acids present in the oils, it is also important to know their antioxidant activity and their stability against lipid oxidation [18]. Oil lipid oxidation is one of the main causes of the loss of quality in color, taste, aroma and nutritional value due to the degradation of essential fatty acids and the production of toxic compounds that can contribute to the development of cancer, atherosclerosis, heart disease and allergic responses [28]. The oxidation process depends mainly on exposure to light, the temperature, the presence of oxygen, the composition of fatty acids and the composition of antioxidant compounds such as polyphenols and tocopherols [29,30]. Bozan et al. [31] found that a high polyunsaturated content of linolenic acid and long-chain fatty acids reduces the oxidative stability of the oils. In addition, Redondo et al. [32] studied the relationship between the lipid composition and oxidative stability of 22 different types of fats and observed that a higher oxidative stability correlates with a lower SFA content. 

Commonly used methods to determine the oxidative stability of lipids include the determination of the peroxide value; the oxidative stability index; the determination of the induction time in the Rancimat test; the determination of the amount of aldehydes formed (TBA method) such as lipid oxidation by-products; and the 2,2-diphenyl-1-picrylhydrazyl (DPPH) free-radical scavenging method [33,34]. In addition, kinetic methods are used in conjunction with these methods to evaluate the degradation process that lipids undergo [35].

Therefore, to study the possible valorization of *Prunus* seeds as functional ingredients, the present work aims to perform a nutritional analysis of the seeds of *P. armeniaca*, *P. persica*, *P. avium* and *P. domestica*, together with the extraction and characterization of the oils obtained from the mentioned seeds in terms of the fatty acid profile, lipid quality indexes and antioxidant activity.

## 2. Results and Discussion

### 2.1. Evaluation of the Nutritional Analysis of Prunus Seeds

The characterization of the approximate composition of peach, apricot, plum and cherry seeds is presented in Table 1. It was performed in terms of the moisture, ash, fat, crude fiber, protein and carbohydrates content, which allowed us to estimate the nutritional value and quality of seed by-products and, thus, their potential applicability as functional ingredients.

As it is presented in Table 1, significant variations (*p*-value < 0.05) among the samples studied were observed in the seed-to-whole stone ratio, moisture, ash, crude fiber, protein nitrogen and carbohydrates contents. These differences were highly likely due to the plants’ genotype, but also to their geographical origin and fruit maturity stages. However, two statistically different groups were observed in the fat content of the seeds: the first group contained peach seeds and the other group contained apricot, plum and cherry seeds.

As can be seen in Table 1, all *Prunus* seeds showed low moisture content, between 3.6 and 7.6% (w), which is beneficial for storage over long periods of time, as they are not susceptible to microorganism attack [36]. In addition, it is noteworthy that every single seed studied was characterized as a suitable source of fat, with percentages between 30 and 38% (w) over the whole seed, followed by protein (10–35%, w), crude fiber (7–24%, w) and carbohydrates (13–25%, w). In line with the results shown in Table 1, kernels are known to be particularly rich in oil [37]. In fact, the seeds with the highest lipid content were apricot (38 ± 2%, w), plum (37.4 ± 0.4%, w) and cherry (36.0 ± 0.2%, w), forming a homogeneous group statistically richer in fat content (*p*-value < 0.05) compared to peach seeds (30 ± 3%, w). The fact that seeds are rich in oils is highly relevant in the food and cosmetic industries, making it possible to recover this waste [38,39]. On the other hand, the presence of a high protein content is of vital importance for the strengthening and maintenance of the muscles and bones that make up the human body, thus providing a high energy intake [40]. In addition, the high content of crude fiber and carbohydrates in seeds display several benefits, such as the reduction of cholesterol, diabetes, coronary heart disease and even the prevention and/or treatment of obesity [41,42,43], showing the high quality and the potential of these bio-residues as functional ingredients [37]. The obtained results from the approximate analysis are comparable with the results of other researchers [37,44,45,46,47,48], which found that *Prunus* seeds were characterized by a protein content of 6–20% (w), a crude fiber content of 15–20% (w), an oil content of 11–57% (w), a moisture content of 5–7% (w), an ash content of 1–3% (w) and a carbohydrate content of 18–27% (w).

### 2.2. Evaluation of the Physico-Chemical Quality Characteristics of Prunus Seed Oils

#### 2.2.1. Density Determination

The oil density is an identifying quality that allows the quality and purity of the oil to be determined [7]. Table 2 shows the density values obtained for peach, apricot, plum and cherry seed oils, which ranged from 0.896 to 0.917 g·mL^−1^, and were in agreement with those reported by other authors [7,49]. The highest density was exhibited by the cherry seed oil (0.917 ± 0.005 g·mL^−1^), followed by plum seed oil (0.903 ± 0.002 g·mL^−1^), apricot seed oil (0.897 ± 0.003 g·mL^−1^) and peach seed oil (0.896 ± 0.001 g·mL^−1^). According to the Fisher’s LSD test, three homogeneous groups (*p*-value < 0.05) were observed, determining that peach and apricot oils did not differ significantly from each other at the 95% confidence level, although they diverged significantly from plum and cherry oils. Neagu et al. [50] reported that the density of vegetable oils is conditioned by their fatty acid composition, minor constituents and temperature. Accordingly, the similarity between peach and apricot seed oils can be likely attributed to a similar fatty acid content. Therefore, determining the lipid composition of the oils is necessary.

#### 2.2.2. Fatty Acid Composition

The fatty acid profile of *Prunus* seed oils was determined using gas chromatography coupled to a flame ionization detector (GC-FID) with previous derivatization to their corresponding methyl esters following the method proposed by Lee et al. [51]. For the identification of the fatty acids present in peach, apricot, cherry and plum seed oils, the chromatogram of FAME 37 component SUPELCO standard mixture (Figure S1, Supplementary Materials) and PUFA No. 3 Menhaden oil Ref 47085—standard mix (Figure S2, Supplementary Materials) were registered. For quantification purposes, an internal standard calibration was carried out with the response factor (RF) using tridecanoic acid (C13:0) as an internal standard. The internal standard was added directly (1 mg) before methylation to correct the losses that could occur in the process of fatty acid derivatization to methyl esters. According to data from the literature [52], the recovery of fatty acids after derivatization processes ranges from 85.6 to 114.1%. The response factors (RF) for each fatty acid in the internal standard calibration ranged from 1.069 to 1.132. The limit of quantification (LOQ) was established as 0.33 mg·g^−1^ and the limit of detection (LOD) was 0.1089 mg·g^−1^. As an example, the chromatograms of plum and cherry seed oils can be observed in Figures S3 and S4 (Supplementary Materials), respectively. A total of 10 fatty acids was identified in peach, apricot and plum seed oils: palmitic acid (C16:0), palmitoleic acid (C16:1n7), margaric acid (C17:0), stearic acid (C18:0), cis-vaccenic acid (C18:1n7c), oleic acid (C18:1n9c), linoleic acid (C18:2n6c), α-linolenic acid (C18:3n3), arachidic acid (C20:0) and gondoic acid (C20:1n9). Moreover, two additional saturated fatty acids were identified in the cherry seed oil: behenic acid (C22:0) and lignoceric acid (C24:0). The lipid content was expressed as a percentage (%) considering a response factor (RF) of 1 and the results are indicated in Table 3. As previously reported by Lazos et al. [7], Atik et al. [53] and Perifanova et al. [54], the oils exhibited a high percentage of unsaturated fatty acids (UFA), ranging from 86% (cherry oil) to 92.2% (apricot oil), with oleic (48.6–72.7%) and linoleic acid (16.4–39.3%) being the dominant ones. The content of saturated fatty acids (SFA) present in the oils varied in the range of 7.8% to 14%, with palmitic acid as the main one (5.71–8.1%). Meanwhile, the levels of other fatty acids present in the oils were below 1% (Table 3).

On the other hand, the total saturated fatty acid (SFA) and unsaturated fatty acid (UFA) content of the cherry seed oils differed significantly (*p*-value < 0.05) with the other oils studied. All *Prunus* seed oils were rich in unsaturated fatty acids, which could have multiple positive health effects. The seed oils had a high content of monounsaturated fatty acids (MUFA), with oleic acid (48.6–72.7%) being the main component of the oils, which could exert beneficial effects on the cardiovascular system and on the proper development and/or functioning of the human brain [55]. Seed oils were also characterized based on their outstanding polyunsaturated fatty acid (PUFA) content (16.5–39.5%), particularly defined by a high content of linoleic acid (16.4–39.3%), which is essential for a healthy diet and for cell membrane development [19]. However, although the saturated fatty acid content represented the minority in all oils, a slight increase was observed in cherry seed oil with a value of 14 ± 2%. In the present study, the PUFA/SFA ratio in the seed oils was especially high (1.86–5.046%), indicating that the oils were healthy, and that they contained an adequate proportion of healthy fatty acids.

**Figure 1:**
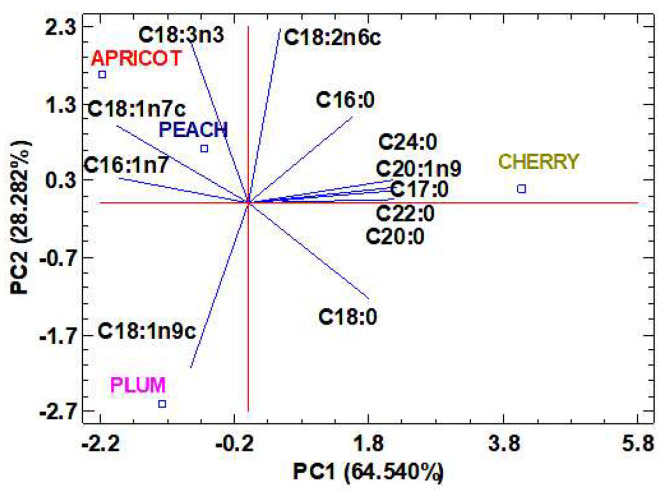
Two−dimensional plot of the principal component analysis (PCA) of the fatty acids present in peach, apricot, plum and cherry seed oils.

To evaluate the fatty acid content relationships with peach, apricot, plum and cherry seed oils, a principal component analysis (PCA) was performed. The results of the PCA analysis are shown in the graph depicted in Figure 1. Two principal components with eigenvalues greater than or equal to 1 accounted for 92.822% of the total data variability. The first principal component (PC1) accounted for 64.540% of the total variability and it was mainly related to margaric acid (C17:0), behenic acid (C22:0), arachidic acid (C20:0), lignoceric acid (C24:0) and gondoic acid (C20:1n9). The second principal component (PC2) explained 28.282% of the total variability and it was defined by the strong correlation with linoleic (C18:2n6c) and α-linolenic acid (C18:3n3). The PCA graph indicated the presence of three homogeneous groups. The first one, formed by peach and apricot seed oils, was characterized by a high content of unsaturated fatty acids such as palmitoleic acid (C16:1n7), cis-vaccenic acid (C18:1n7c) and α-linolenic acid (C18:3n3). The presence of high linolenic acid content in oils can have multiple health benefits, as this essential omega-3 (n-3) polyunsaturated fatty acid plays vital roles in proper brain development and function, cardiovascular health and the anti-inflammatory response [56]. In addition, the presence of high contents of other UFAs such as cis-vaccenic acid and palmitoleic acid has an anti-inflammatory and protective effect against cardiovascular diseases [57,58]. The second group consisted of plum seed oil, which was characterized by a high oleic acid content (C18:1n9c). This type of non-essential fatty acid in the oil can be used in the treatment and prevention of various disorders, such as autoimmune or cardiovascular diseases, metabolic disorders and cancer [55]. The third and last group consisted of cherry seed oil, which is characterized by a high SFA content, especially behenic acid (C22:0), margaric acid (C17:0) and arachidic acid (C20:1n9c). 

Regarding the correlations between the different fatty acids presented in the four different oils (Figure 1), it was observed that the UFA, palmitoleic acid (C16:1n7) and cis-vaccenic acid (C18:1n7c) were highly positively correlated with each other. Palmitoleic acid was correlated negatively with the saturated fatty acids margaric acid (C17:0), behenic acid (C22:0) and arachidic acid (C20:0); and the unsaturated fatty acid gondoic acid (C20:1n9). However, cis-vaccenic fatty acid was negatively correlated with stearic acid (C18:0). A high negative correlation was also observed between the two main components of the oils: linoleic acid (C18:2n6c) and oleic acid (C18:1n9c).

#### 2.2.3. Lipid Health Quality Indexes

The atherogenicity index (AI) showed two homogeneous groups (*p*-value < 0.05): on the one hand, peach and cherry seed oil were grouped together, and on the other, apricot and plum seed oil were grouped together. All oils showed low values of this index, where the lowest value was found in plum seed oil (0.0626) and the highest in cherry seed oil (0.09). The results corresponding to the thrombogenicity (TI) index showed that peach, apricot and plum seed oils were statistically similar (*p*-value ≥ 0.05), while cherry seed oil differed from them. The lowest value corresponded to the apricot kernel oil (0.166) and the highest to the cherry kernel oil (0.28). According to the literature [23,24,59], low values of both the AI and TI are related to the prevention of coronary heart disease. Thus, the lower these values, the healthier the food, indicating the promising potential of these seed oils.

According to the desirable fatty acid (DFA) index, three statistically homogeneous groups were found (peach and apricot oil, apricot and plum oil and, lastly, cherry oil). High values (90–94) were observed for all four *Prunus* seed oils (Table 4), evidencing their high hypocholesterolaemic properties. The hypocholesterolemic/hypercholesterolemic (H/H) ratio correlates with the DFA index and measures the bioactive properties of oils to lower blood cholesterol levels [60]. In this case, the oils were grouped into two statistically homogeneous groups and showed high values for this nutritional index (11.3–15.83). 

On the other hand, all seed oils were shown to have a higher proportion of omega-6 fatty acids than omega-3 fatty acids (Table 4). The oils presented significant differences among them (*p*-value < 0.05), with the lowest value found in plum seed oil (201.5 ± 0.6). Although these values suggest a health risk, the presence of high amounts of linoleic acid is required by the human body for the maintenance of cell membranes, brain function and the transmission of nervous impulses in normal conditions and the correct oxygenation of the blood [61]. Therefore, a balanced omega-6/omega-3 ratio diet is needed.

Considering all the nutritional lipid indexes (AI, TI, H/H, DFA, n6/n3 ratio) used to evaluate the quality of the oils, plum seed oil was the one that presented the best nutritional quality. However, all *Prunus* seed oils could play beneficial roles in human health, particularly in the prevention and/or treatment of coronary and cardiovascular diseases and obesity [62,63]. Due to the interesting nutritional characteristics of *Prunus* seed oils, they could be used in the food and/or nutraceutical industries as natural food supplements or additives [37,39].

#### 2.2.4. Antioxidant Activity

The determination of the oil antioxidant activity is another parameter that is used to evaluate the quality of oils, since this bioactive property correlates with the lipid composition and the presence of natural antioxidants in the oil [64]. Table 5 shows the antioxidant activity of peach, apricot, plum and cherry seed oils (expressed as mg·mL^−1^ of oil). Trolox was used as a standard and its IC_50_ was 0.0025 ± 0.0001 mg·mL^−1^. All studied oils showed high values of IC_50_, ranging from 20 to 35 mg·mL^−1^, with respect to the Trolox standard. Furthermore, the IC_50_ values obtained for *Prunus* seed oils were higher than those reported by Fratianni et al. [38], indicating the interesting potential of the oils obtained from the agri-food waste. Apricot and plum oils were those with the highest antioxidant capacity (IC_50_ = 20–21 mg·mL^−1^), compared to peach and cherry oils, which were characterized by a significantly lower (*p*-value < 0.05) antioxidant capacity (IC_50_ = 31–35 mg·mL^−1^).

**Figure 2:**
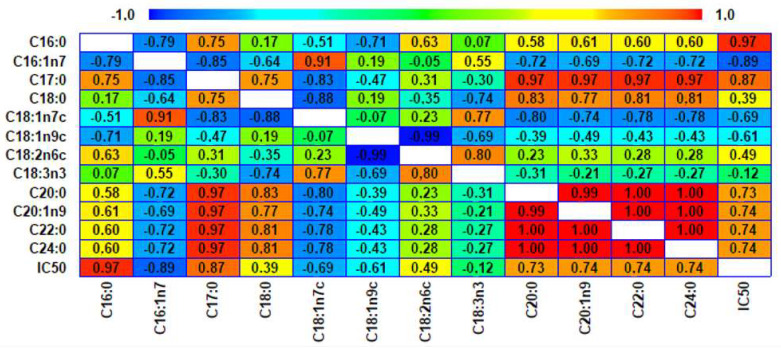
Pearson correlation heat map between fatty acid contents and antioxidant activity of *Prunus* seed oils. IC_50_ values mean the concentration of sample required to scavenge 50% of DPPH free radical.

To establish the possible relationship between the fatty acid composition (Table 3) and the antioxidant capacity of seed oils, a Pearson correlation analysis was carried out (Figure 2). The correlation coefficients ranges from −1 to +1 to express the linear relationship between the different study factors. The correlation analysis showed that the main fatty acids affecting the oils’ antioxidant capacity (IC_50_ value) were palmitic acid (C16:0) and palmitoleic acid (C16:1n7). A high positive correlation was also observed between the content of palmitic acid and the IC_50_ value (r = 0.97). A higher content of palmitic acid in the oil led to an increase in the IC_50_ value and, therefore, a decrease in the antioxidant capacity. Palmitic acid is associated with increasing total cholesterol levels in plasma [38]; however, it was found in low proportions in oils (5.71–8.1%). Conversely, a high negative correlation was observed between the IC_50_ value and the palmitoleic acid (C16:1n7) content (r = −0.89), indicating that the high content of this type of UFA in the oils contributes positively to the antioxidant capacity (lowest IC_50_ value) and acts beneficially in the human body, protecting against oxidative stress [55]. 

### 2.3. Comparative Study of the Oxidative Stability of Seed Oils

Lipid oxidation is one of the biggest problems affecting oil quality. Therefore, the study of the oxidative stability is required.

The oil oxidative stability was measured over a period of 1–22 days using the 2,2-diphenyl-1-picrylhydrazyl (DPPH) free-radical scavenging method. The results obtained are shown in Table 6.

**Figure 3:**
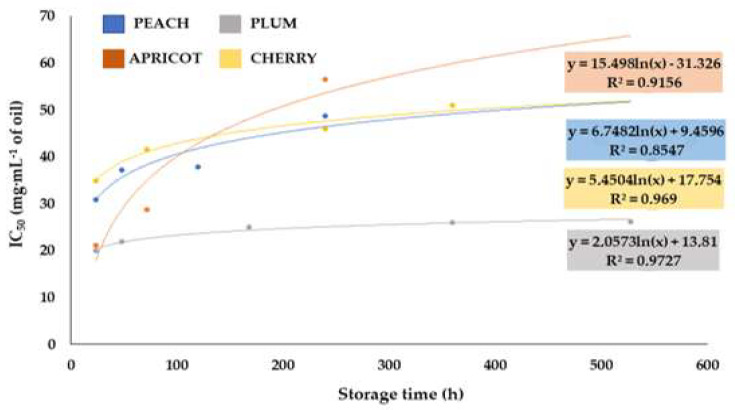
Kinetics curves of logarithmic model of IC_50_ values versus storage time of peach, apricot, plum and cherry seed oils.

To evaluate the trend of the oxidative stability, IC_50_ values were plotted versus storage time (Figure 3). The curves shown in Figure 3 were non-linear and fitted adequately to a logarithmic model (with correlation coefficients (R^2^) between 0.8547 and 0.9727). Thus, the variation in antioxidant capacity during storage time followed a logarithmic model of the first-order degradation kinetic reaction [35,65]. In the logarithmic curve of plum oil, an apparently constant trend was observed between the IC_50_ values and the storage times, indicating its high stability against lipid oxidation. However, in apricot, peach and cherry seed oils, an increasing trend of the IC_50_ value was observed at 240 h (22 days) of storage time, which indicated that the lipid oxidation phenomenon had occurred.

To determine the time at which the oil was stable against the oxidation of its fatty acids the t_1/2_ value was estimated, which refers to the time at which the concentration of oil antioxidants is reduced by half. For this purpose, the IC_100_ was calculated as the sample concentration required to remove 100% of the free radical DPPH. From this value, ln(100-IC_100_) (ln_c.antioxidants_) was calculated and plotted against storage time, following the first-order linear kinetic equation (ln_c.antioxidants_ = ln_C0_ − kt). The data related to the linear fit (intercept, slope and correlation coefficient (R^2^)) are given in Table 7. 

It was observed that the oxidative stability of all oils conformed to first-order kinetics with high correlation factors (R^2^) ranging from 0.78 to 1.00. The times at which the antioxidant concentration halved were different for each oil. On the one hand, the longest time and, thus, the highest stability against lipid oxidation, was observed for the plum oil (t_1/2_ = 1732 h (72 days)). On the other hand, peach oil showed the shortest stability time (t_1/2_ = 58 h (2 days)). Apricot kernel oil and cherry oil showed t_1/2_ values of 110 and 121 h (4 and 5 days), respectively.

**Figure 4:**
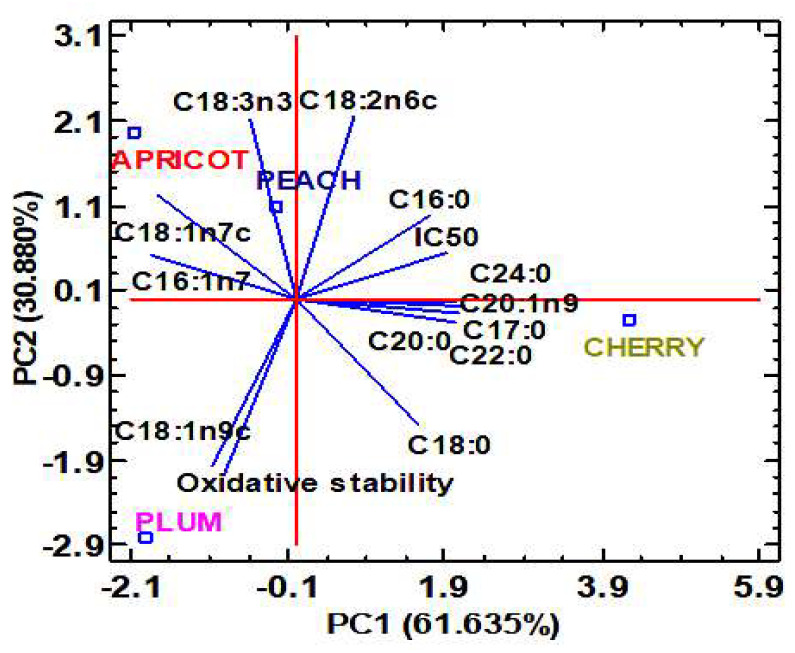
Two-dimensional principal component analysis (PCA) plot of fatty acid content, antioxidant activity (IC_50_) and oxidative stability of peach, apricot, plum and cherry seed oils.

The results obtained according to literature data [32,61] evidenced that the oil composition strongly affects its antioxidant activity and oxidative stability. Consequently, the effect of fatty acids on oil oxidative stability was studied using principal component analysis based on the stability time data reported in Table 7 and the data concerning fatty acid composition (Table 3). Two principal components, with eigenvalues greater than or equal to 1, were extracted. They accounted for 93.213% of the total data variability (Figure 4).

The first principal component (PC1) represented 60.401% of the total data variability and it was mainly related to margaric acid (C17:0), behenic acid (C22:0), arachidic acid (C20:0), lignoceric acid (C24:0) and gondoic acid (C20:1n9). The second principal component (PC2) explained 32.813% of the total data variability and it was defined by the strong correlation with both linoleic (C18:2n6c) and α-linolenic acids (C18:3n3). In the PCA graph depicted in Figure 4, a high positive correlation was also observed between the oxidative stability and the oleic acid content. On the contrary, the maximum negative correlation was observed between the oxidative stability and the content of linoleic acid. Similarly, Nederal et al. [66] observed that the oxidative stability of cold-pressed pumpkin oils was positively correlated with the oleic acid content and negatively correlated with the linoleic and α-linolenic acid contents. This fact could be explained by the presence of consecutive double bonds, which are more susceptible to attack by the oxygen present in the environment. Furthermore, it was observed that plum seed oil had the greatest stability against the oxidation of fatty acids due to its high content of oleic monounsaturated fatty acid. 

The adequate antioxidant activity and oxidative stability of *Prunus* seed oils opens new fields of application in the cosmetic industry for their use as active ingredients in antioxidant cosmetics [39].

## 3. Materials and Methods

### 3.1. Reagents and Solvents

Analytical grade reagents were required in the procedures. *n*-Hexane (96%) and methanol (MeOH, ≥99%) for HPLC gradient quality were supplied by Scharlab (Barcelona, Spain). Sodium hydroxide pellets (NaOH) (98%), sulphuric acid (H_2_SO_4_, 98%), boric acid (≥99%) and methylene blue (82%) were obtained from Panreac (Barcelona, Spain). Dimethyl sulfoxide (DMSO, ≥99.9%) and 2,2-diphenyl-1-picrylhydrazyl (DPPH, ≥99.9%) were provided by Sigma-Aldrich (St. Louis, MO, USA). Methyl red, Kjeldahl catalyst tables and distilled water system were purchased from Merck (Madrid, Spain). Fatty acid standards FAME 37 component SUPELCO Ref CRM47885, PUFA No. 3 Menhaden oil Ref 47085-U and tridecanoic acid (C13:0, ≥ 99%) were purchased from Sigma-Aldrich (Barcelona, Spain). The standard Trolox was provided by Sigma-Aldrich (Burghasen, Germany).

### 3.2. Sample Preparation

Plum (*Prunus domestica*) and cherry (*Prunus avium*) pits were purchased from The Jerte Valley Cooperatives Group (Cáceres, Spain). Peach pits (*Prunus persica*) and apricot pits and seeds (*Prunus armeniaca*) were purchased from The Agri-Food Cooperatives of Castilla La Mancha (Hellín, Albacete). 

Prior to any treatment, fresh samples, received during the campaign period corresponding to the year 2022, were air-dried at 40 °C (Digitheat oven, J.P Selecta^®^, Abrera, Barcelona, Spain) for 24 h, and then stored at room temperature until use.

For compositional analysis, the stones of the *Prunus* fruits studied were separated manually, using a hammer, into shell and seed. The seeds were then crushed in an ultra-centrifugal grinder (Retsh™ ZM200, Haan, Alemania) and sieved with a stainless-steel sieve to particle sizes below 1 mm. Seed samples were stored in clear plastic zip-lock bags until analysis.

### 3.3. Nutritional Analysis of the Four Types of Fruit Seed of Prunus Family

#### 3.3.1. Determination of Seed-to-Whole Stone Ratio

The percentage in weight of seeds in relation to the stone was determined by weighing 25 stones and their respective seeds on a 0.0001 g precision digital analytical balance (Precisa Series 290, Dietikon, Switzerland) for each of the *Prunus* varieties. 

#### 3.3.2. Determination of Moisture Content

The moisture content of the seeds was determined according to the standard procedure AOAC 925.10 [67], with slight modifications. Approximately 2 g of seed sample was weighed on a dried crucible and introduced in an oven at 105 °C for 2 h and 30 min until a constant weight was obtained. After cooling, the crucible was weighed again and the free water content was calculated as sample weight loss and expressed as a percentage. It was determined as percentage in weight (mean ± standard deviation, n = 3).

#### 3.3.3. Determination of Ash Content

For the determination of ash, AOAC 923.03 standard procedure was followed [68]. Briefly, a sample amount of 2 g was weighed on a clean and dry crucible, which was then placed in muffle furnace (J.P Selecta^®^, Abrera, Barcelona, Spain) at 550 °C for 4 h. The appearances of grey/white ash indicated the complete oxidation of all organic matter in the sample. Then, the crucible with the ash was cooled in the desiccator and weighed on a precision digital analytical balance (Precisa Series 290, Dietikon, Switzerland) until reaching a constant weight. Finally, the ash content was then calculated as a percentage in weight of dried sample and expressed as mean ± standard deviation (n = 3). 

#### 3.3.4. Determination of Fat Content

The total oil content in the samples was determined following an AOAC 960.39 procedure [69]. First, a Soxhelt extraction was carried out by placing 15 g of seed with a particle size of less than 1 mm into a cellulose cartridge (FILTER-LAB^®^, Darmstadt, Germany), within a collecting flask of 250 mL capacity (Mervilab S.A., Madrid, Spain) that was previously dried in an oven (Digitheat, J.P Selecta^®^, Abrera, Barcelona, Spain) at 105 °C for 2 h and weighed. The cellulose cartridge was inserted into the Soxhlet extractor body and 150 mL of *n*-hexane (seed-solvent ratio 1:10 (*w*/*v*)) was added to the collecting flask. *Prunus* seeds were then extracted under reflux at 69 °C for 6 h (6–8 cycles/h). Then, the solvent was removed using a rotary evaporator (Buchi™ Rotavapor™ R-100, Fisher Scientific, Hampton, VA, USA) at 69 °C, and the collecting flask with the extract was placed in a vacuum oven (Vaciotem-TV, digital, J.P Selecta^®^, Abrera, Barcelona, Spain) at 40 °C for 24 h. Finally, it was cooled in a desiccator for 30 min and weighed again. This procedure was carried out in triplicate. The oils were stored in airtight amber-colored glass bottles and kept at 4 °C prior to analysis. The percentage of fat was expressed as mean ± standard deviation (n = 3) on a dry basis.

#### 3.3.5. Determination of Crude Fiber Content

The content of crude fiber in the different seed samples was assessed according to an official methodology described elsewhere [70]. For this purpose, approximately 2 g of defatted seed was weighed on a precision digital analytical balance and subjected to a boiling step for 30 min with 200 mL of 0.128 M H_2_SO_4_ solution. Then, the solution was filtered through a cotton filter to drain the acid and the solid residue was washed with hot distilled water and subjected to a further boiling for 30 min with 200 mL of 0.313 M NaOH solution. Following filtration through a cotton filter, the insoluble residue was dried in an oven (Digitheat, J.P Selecta^®^, Abrera, Barcelona, Spain) at 230 °C for 2 h and weighed, and subsequently placed in a muffle furnace (J.P Selecta^®^, Abrera, Barcelona, Spain) at 550 °C for 2 h. Finally, the crucible with the samples was cooled in the desiccator and weighed again. The crude fiber content was determined using the quotient of the difference in weight of the dry insoluble residue and the incinerated residue between the total mass of the dry seed. This test was performed in triplicate and expressed as mean ± standard deviation.

#### 3.3.6. Determination of Protein Nitrogen Content

The protein content of the seed samples was determined using the Kjeldahl method [71]. Approximately 1 g of dried sample was added in digestion flask with 12 mL of concentrated H_2_SO_4_ and one Kjeldahl catalyst tablet (Merck, Darmstadt, Germany). The mixture was placed in the digestion module (VELP Scientifica, Usmate, Italy) at 410 °C for 1 h. After cooling, the digested mixture was placed in the steam distillation module (VELP Scientifica, Usmate, Italy) with 50 mL of 2% (w) boric acid, two drops of methyl red and one drop of methylene blue. The system was programmed to add 18 mL of 40% (w) NaOH in 12 s and the distillation process took place for 210 s. Finally, the distilled ammonia collective in the boric acid solution was titrated with 0.03 M H_2_SO_4_ solution until it turned green to violet. The procedure was carried out in triplicate and two blanks were also prepared. Finally, the protein content was estimated from the percentage of nitrogen using Equation (1), where the coefficient of 6.25 is the correction factor for this type of sample and “%N” is the percentage of nitrogen in the sample; and Equation (2) where “s” is the sample titration reading, “b” is the blank titration reading, “M” is the molarity of H_2_SO_4_ and “0.014” the milli equivalent weight of nitrogen.

(1) Proteinnitrogen(%)=6.25×%N

(2) $$N(\%)=\frac{(s-b)\times 0.014\times M}{{wt}_{sample}}$$

#### 3.3.7. Determination of Total Carbohydrates

The carbohydrate content in *Prunus* seeds, expressed as the mean value of the weight percentage ± standard deviation (n = 3), was calculated by subtracting the sum of the percentage of moisture, ash, fat and protein nitrogen contents from 100% according to Ayoola et al. [72].

### 3.4. Physico-Chemical Characterization of Prunus Seed Oils

#### 3.4.1. Determination of Oil Density

The density of oils was determined using a density meter (Anton Paar DMA 5000, Madrid, Spain) with an operative temperature of 25 °C. The tests were carried out in triplicate for each sample. 

#### 3.4.2. Determination of Fatty Acid Profile

The determination of fatty acid composition in the extracted oils was carried out using gas chromatography coupled to a FID detector (Agilent Technologies, 7820A, Santa Clara, CA, USA), following the internal derivatization procedure to methyl esters proposed by Lee et al. [51]. Prior to the chromatographic analysis, the extracted oils (200 mg approximately) were freeze-dried (Sp Scientific, Warminster, PA, USA) at −50 °C and 150 mbar for 72 h. To synthesize fatty acid methyl esters (FAME), the fatty acids were incubated with 0.5 M sodium methoxide at 60 °C for 15 min, followed by extraction with acetyl chloride in methanol 1:10 (*v*/*v*) and incubation at 60 °C for 60 min. One milliliter of purified water, 1.5 mL of hexane and sodium sulphate was added to each sample, followed by a centrifugation step (4 °C, 1500 rpm for 5 min). One milliliter of the supernatant was used for subsequent analyses. Samples were evaporated in a Savant™ SPD131DDA SpeedVac™ concentrator (Thermo Fisher Scientific, Madrid, Spain) and resuspended in hexane (Thermo Fisher Scientific, Madrid, Spain) The chromatographic separation was achieved using a capillary chromatographic column (60 m × 250 μm × 0.25 μm) (Agilent Technologies DB-23 Ref 122-2362), helium as a carrier gas at 1 mL·min^−1^ flow rate and split injection mode (40:1). The detector temperature was set at 260 °C and the injector furnace temperature at 250 °C. The oven temperature gradient started at 100 °C for 2 min, after which the temperature increased by 8 °C/min to 145 °C. This condition was maintained for 20 min and then rose by 5 °C/min to 230 °C. Chromatograms were recorded and analyzed using EZChrom Elite compact 3.3.2. software (Agilent Technologies, Madrid, Spain). 

The identification of the fatty acids was performed by comparing the retention time of the samples with that of the commercial standards (FAME 37 SUPELCO Ref CRM47885 + PUFA No. 3 Menhaden oil Ref 47085-U). For quantification, an internal standard calibration of response factors (RF) was performed as a way to correlate the ratio of areas between each FAME and the internal standard with the FAME concentration, using tridecanoic acid (C13:0) as internal standard (1 mg was added before methylation). To determine the relative amounts of each FAME present in the different samples, a response factor of 1 was set and the composition was expressed as a percentage (%) considering the individual area of each FAME and the total area (considering that 100% corresponds to the sum of all the areas of the determined analytes). Analyses were performed in duplicate.

#### 3.4.3. Lipid Nutritional Quality Indexes

To estimate the nutritional value of the seed oils studied the total content of saturated (SFA), unsaturated (UFA), monounsaturated (MUFA) and polyunsaturated (PUFA) fatty acids, along with the lipid nutritional quality indexes related to the fatty acid profile and healthy fat consumption (the atherogenicity index (AI), thrombogenicity index (TI), hypocholesterolemic-hypercholesterolemic ratio (H/H) and desirable fatty acid (DFA) index) were calculated as defined elsewhere [24,25]:

(3) ∑SFA=C16:0+C17:0+C18:0+C20:0+C22:0+C24:0

(4) ∑UFA=∑MUFA+∑PUFA

(5) ∑MUFA=C16:1n7+C18:1n7c+C18:1n9c

(6) ∑PUFA=C18:2n6c+C18:3n3+C20:1n9

(7) DFA=∑MUFA+∑PUFA+C18:0

(8) $$AI=\frac{C12:0+4(C14:0)+C16:0}{\sum MUFA+\sum PUFA}$$

(9) $$H/H=\frac{C18:1+\sum PUFA}{C14:0+C16:0}$$

(10) $$TI=\frac{C12:0+C16:0+C18:0}{\left(0.5\sum MUFA\right)+\left(0.5\times n6\sum PUFA\right)+\left(3\times n3\sum PUFA\right)\times (\frac{n3\sum PUFA}{n6\sum PUFA})\;}$$

where n3∑PUFA stands for the sum of omega-3 polyunsaturated fatty acids, n6∑PUFA is the sum of omega-6 polyunsaturated fatty acids, C16:0 corresponds to palmitic acid, C16:1n7 is palmitoleic acid, C17:0 is margaric acid, C18:0 is stearic acid, C18:1n7c is cis-vaccenic acid, C18:1n9c is oleic acid, C18:2n6c is linoleic acid, C18:3n3 is α-linolenic acid, C20:0 is arachidic acid, C20:1n9 is 11-eicosenoic acid, C22:0 is behenic acid and C24:0 is lignoceric acid.

Finally, the n6/n3 fatty acid ratio, which is related to a healthy diet, was calculated according to the equation proposed by Asha et al. [73]. 

#### 3.4.4. Determination of the Antioxidant Capacity of Seed Oils

The antioxidant activity of *Prunus* seed oils was measured as DPPH free-radical scavenging capacity, following the methodology proposed by Fratianni et al. [38], which was slightly modified. Briefly, 30 μL of eight methanolic working solutions (0–30 μL) prepared using dilution from oil solutions (448–850 mg·mL^−1^, DMSO) were mixed with 270 μL of 6 × 10^−5^ M DPPH methanolic solution in 96-well microplate. The final solutions were stored in the dark at room temperature and under shaking (300 rpm) for 60 min. The absorbance was then measured at 515 nm using a Thermo Scientific Multiskan GO spectrophotometer. Finally, the oil concentrations were plotted against DPPH remaining percentages and the results were expressed as IC_50_ values, i.e., the concentration of sample required to inhibit 50% the initial DPPH concentration. The assay was performed in duplicate and Trolox standard was used as positive control.

#### 3.4.5. Evaluation of the Antioxidant Stability of Seed Oils

The antioxidant activity of *Prunus* seed oils was monitored for 22 days to determine the oxidative stability of the evaluated oils using the DPPH method described in Section 3.4.4. 

For this purpose, the IC_100_, i.e., the minimum oil concentration that completely reduced the initial DPPH absorbance at 515 nm, was determined over the storage time (24 h and 22 days). The former was estimated as the initial concentration of antioxidant agents present in the oils and the data obtained were fitted to first-order kinetics, according to Equation (11), where “ln_c.antioxidants_” is the logarithm of the antioxidants present in the oils during storage time, “ln_C0”_ is the logarithm of IC_100_ value, “k” is the velocity constant and “t” corresponds to the storage time.

Thus, from the logarithmic calibration fits, the mean degradation time, known as the time in which the initial concentration of antioxidants is halved, was calculated for the different *Prunus* seed oils.

(11) $${ln}_{c.antioxidants}={ln}_{C0}-kt$$

### 3.5. Statistical Analysis

Statistical analysis was performed using the Statgraphics 19 software package (Statgraphics Technologies Inc., Rockville, MD, USA). Data were statistically analyzed using multivariate and univariate analysis of variance (ANOVA), principal component analysis (PCA) and Pearson correlation analysis. Significant differences between determinations were evaluated using Fisher’s least significant difference (LSD) test at 95% confidence level (*p*-value < 0.05).

## 4. Conclusions

This study focuses on the valorization of peach, apricot, plum and cherry seed agri-food waste based on the extraction of seed oil and its characterization in terms of fatty acid content, antioxidant activity and oxidative stability. 

*Prunus* seeds presented high added value due to their remarkable fat (30–38%, w), crude fiber (7–24%, w), protein (10–35%, w) and carbohydrate (13–25%, w) contents. The main components of interest of the seeds were the seed oils. The high content of unsaturated fatty acids in the oils, such as oleic (48.6–72.7%) and linoleic acid (16.4–39.3%), and their adequate heart-healthy indexes make their potential use in the food and nutraceutical industry feasible, either as food supplements or nutraceuticals. In addition, the outstanding antioxidant activity (IC_50_ = 30–35 mg·mL^−1^) and the high oxidative stability during 1–22 days of storage of the oils, especially the plum seed oil, opens new applications in the cosmetic industry as an active ingredient in antioxidant cosmetics.

## Figures and Tables

**Table 1:** Nutritional analysis of peach, apricot, plum and cherry seeds.

Sample	Seed (%, w)	Moisture (%, w)	Ash (%, w)	Fat (%, w)	Crude Fiber (%, w)	Protein Nitrogen (%, w)	Carbohydrates (%, w)
Peach	2.4 ± 0.3 ^a^	7.6 ± 0.4 ^a^	6.1 ± 0.1 ^a^	30 ± 3 ^a^	8.63 ± 0.02 ^a^	35 ± 2 ^a^	13 ± 3 ^a^
Apricot	12.4 ± 0.6 ^b^	5.8 ± 0.3 ^b^	4.8 ± 0.4 ^b^	38 ± 2 ^b^	7.49 ± 0.02 ^b^	28.7 ± 0.8 ^b^	15 ± 3 ^b^
Plum	9.5 ± 0.3 ^c^	4.7 ± 0.4 ^c^	2.16 ± 0.02 ^c^	37.4 ± 0.4 ^b^	21.4 ± 0.2 ^c^	16 ± 2 ^c^	18 ± 2 ^c^
Cherry	10.3 ± 0.5 ^d^	3.6 ± 0.1 ^d^	1.43 ± 0.02 ^d^	36.0 ± 0.2 ^b^	23.9 ± 0.6 ^d^	10.3 ± 0.7 ^d^	25 ± 1 ^d^

Values are expressed as mean ± standard deviation (n = 3), in dry weight. Values followed by a different superscript in a column differ significantly (*p*-value < 0.05) according to ANOVA and Fisher’s LSD test.

**Table 2:** Density values of peach, apricot, plum and cherry seed oils.

Sample	Density (g·mL^−1^)
Peach oil	0.896 ± 0.001 ^a^
Apricot oil	0.897 ± 0.003 ^a^
Plum oil	0.903 ± 0.002 ^b^
Cherry oil	0.917 ± 0.005 ^c^

Data obtained are expressed as mean ± standard deviation (n = 3). Values with different letters in the same column denote significant differences (*p*-value < 0.05) among samples according to ANOVA and Fisher’s LSD test.

**Table 3:** Fatty acid composition of peach, apricot, plum and cherry seed oils.

Fatty Acids	Peach Oil (%)	Apricot Oil (%)	Plum Oil (%)	Cherry Oil (%)
Palmitic acid (C16:0)	(7.95 ± 0.08) ^a^	(6.36 ± 0.03) ^b^	(5.71 ± 0.03) ^b^	(8.1 ± 0.8) ^a^
Palmitoleic acid (C16:1n7)	(0.579 ± 0.002) ^a^	(1.10 ± 0.03) ^b^	(0.81 ± 0.01) ^c^	(0.39 ± 0.01) ^d^
Margaric acid (C17:0)	(0.0612 ± 0.0008) ^a^	(0.051 ± 0.002) ^b^	(0.0529 ± 0.0001) ^a,b^	(0.092 ± 0.007) ^c^
Stearic acid (C18:0)	(1.401 ± 0.004) ^a^	(1.27 ± 0.05) ^a^	(2.86 ± 0.05) ^b^	(3.8 ± 0.6) ^c^
Cis-Vaccenic acid (C18:1n7c)	(1.302 ± 0.007) ^a^	(1.863 ± 0.024) ^b^	(1.185 ± 0.005) ^c^	(0.71 ± 0.02) ^d^
Oleic acid (C18:1n9c)	(52.9 ± 0.4) ^a^	(49.6 ± 0.5) ^b^	(72.7 ± 0.2) ^c^	(48.6 ± 0.9) ^b^
Linoleic acid (C18:2n6c)	(35.4 ± 0.3) ^a^	(39.3 ± 0.5) ^b^	(16.4 ± 0.2) ^c^	(36.1 ± 0.9) ^a^
α-Linolenic acid (C18:3n3)	(0.129 ± 0.006) ^a^	(0.172 ± 0.008) ^b^	(0.081 ± 0.001) ^c^	(0.1063 ± 0.0003) ^d^
Arachidic acid (C20:0)	(0.143 ± 0.006) ^a^	(0.14 ± 0.01) ^a^	(0.1952 ± 0.0004) ^a^	(1.3 ± 0.3) ^b^
Gondoic acid (C20:1n9)	(0.090 ± 0.002) ^a^	(0.104 ± 0.005) ^b^	(0.079 ± 0.002) ^c^	(0.398 ± 0.001) ^d^
Behenic acid (C22:0)	ND	ND	ND	(0.23 ± 0.04)
Lignoceric acid (C24:0)	ND	ND	ND	(0.18 ± 0.02)
∑SFA	(9.55 ± 0.09) ^a^	(7.8 ± 0.1) ^a^	(8.82 ± 0.02) ^a^	(14 ± 2) ^b^
∑UFA	(90.45 ± 0.09) ^a^	(92.2 ± 0.1) ^a^	(91.18 ± 0.02) ^a^	(86 ± 2) ^b^
∑MUFA	(54.9 ± 0.4) ^a^	(52.7 ± 0.6) ^b^	(74.7 ± 0.2) ^c^	(50.1 ± 0.9) ^d^
∑PUFA	(35.5 ± 0.3) ^a^	(39.5 ± 0.5) ^b^	(16.5 ± 0.2) ^c^	(36.2 ± 0.9) ^a^
PUFA/SFA ratio	(3.7198 ± 0.0001) ^a^	(5.046 ± 0.003) ^b^	(1.86 ± 0.03) ^a^	(2.7 ± 0.4) ^c^

Results are the mean of two replicates, expressed in percentages with estimates of standard deviation. Values on the same row with different letters denote significant differences (*p*-value < 0.05) among samples according to ANOVA and Fisher’s LSD test. ND: undetectable. SFA: saturated fatty acids. UFA: unsaturated fatty acids. MUFA: monounsaturated fatty acids. PUFA: polyunsaturated fatty acids (unsaturation ≥ 2). PUFA/SFA: ratio between polyunsaturated and saturated fatty acids.

**Table 4:** Indexes of the nutritional quality of peach, apricot, plum and cherry seed oils.

Lipid Indexes	Peach Oil	Apricot Oil	Plum Oil	Cherry Oil
Desirable fatty acid (DFA)	(91.85 ± 0.09) ^a^	(93.44 ± 0.5) ^a,b^	(94.05 ± 0.03) ^b^	(90 ± 1) ^c^
Atherogenicity (AI)	(0.088 ± 0.001) ^a^	(0.0691 ± 0.0004) ^b^	(0.0626 ± 0.0003) ^b^	(0.09 ± 0.01) ^a^
Thrombogenicity (TI)	(0.207 ± 0.002) ^a^	(0.166 ± 0.002) ^a^	(0.188 ± 0.001) ^a^	(0.28 ± 0.04) ^b^
Hypocholesterolemic/Hypercholesterolemic (H/H)	(11.3 ± 0.1) ^a^	(14.29 ± 0.08) ^b^	(15.83 ± 0.06) ^b^	(11 ± 1) ^a^
n6/n3 fatty acid ratio	(275 ± 10) ^a^	(228 ± 8) ^b^	(201.5 ± 0.6) ^c^	(339 ± 9) ^d^

Values are expressed as mean ± standard deviation (n = 2). Values with different letters in the same row denote significant differences (*p*-value < 0.05) among samples according to ANOVA and Fisher’s LSD test. n-6/n-3: ratio between omega-6 and omega-3 fatty acids.

**Table 5:** Antioxidant activity determined after 24 h storage of peach, apricot, plum and cherry kernel oils.

Oils	DPPH IC_50_ (mg·mL^−1^ of Oil)
Peach oil	(31 ± 3) ^a^
Apricot oil	(21.2 ± 0.9) ^b^
Plum oil	(20 ± 3) ^b^
Cherry oil	(35 ± 4) ^a^

Values are expressed as mean ± standard deviation (n = 2). Values with different letters denote significant differences (*p*-value < 0.05) among samples according to ANOVA and Fisher’s LSD test. IC_50_ values are the concentration of sample required to scavenge 50% of 2,2-diphenyl-1-picrylhydrazyl (DPPH) free radical.

**Table 6:** Variation in the antioxidant capacity of peach, apricot, plum and cherry seed oils with storage time.

Storage Time (h)	IC_50_ Peach Oil (mg·mL^−1^ of Oil)	IC_50_ Apricot Oil (mg·mL^−1^ of Oil)	IC_50_ Plum Oil (mg·mL^−1^ of Oil)	IC_50_ Cherry Oil (mg·mL^−1^ of Oil)
24 (1 day)	(31 ± 3) ^a^	(21.2 ± 0.9) ^a^	(20 ± 3) ^a^	(35 ± 4) ^a^
48 (2 days)	(37 ± 1) ^b^	n.m.	(21.9 ± 0.7) ^a,b^	n.m.
72 (3 days)	n.m.	(28.7 ± 0.5) ^b^	n.m.	(41.6 ± 0.2) ^a,b^
120 (5 days)	(37.8 ± 0.6) ^b^	n.m.	n.m.	n.m.
168 (7 days)	n.m.	n.m.	(25 ± 1) ^b^	n.m.
240 (10 days)	(39 ± 3) ^c^	(57 ± 1) ^c^	n.m.	(46 ± 1) ^b,c^
360 (15 days)	n.m.	n.m.	(26 ± 3) ^b^	(51 ± 4) ^c^
528 (22 days)	n.m.	n.m.	(26.2 ± 0.3) ^b^	n.m.

Values are expressed as mean ± standard deviation (n = 2). IC_50_ values mean the concentration of sample required to scavenge 50% of DPPH free radical and n.m. signifies not measured on that specific storage time. For each type of oil, values with different letters in the same column denote significant differences (*p*-value < 0.05) between storage times according to ANOVA and Fisher’s LSD test.

**Table 7:** Parameters of the linear fit (intercept, slope, correlation coefficient (R^2^)) of ln_c.antioxidants_ versus storage time of peach, apricot, plum and cherry kernel oils.

Prunus Seed Oil	Intercept (mg·mL^−1^)	Slope (h^−1^)	t_1/2_ (h)	R^2^
Peach	(−0.012 ± 0.003)	(4.0 ± 0.4)	58 (2 days)	0.90
Apricot	(−0.006 ± 0.001)	(4.2± 0.1)	110 (4 days)	1.00
Plum	(−0.0004 ± 0.0001)	(4.0 ± 0.3)	1732 (72 days)	0.78
Cherry	(−0.0057 ± 0.0001)	(3.4 ± 0.2)	121 (5 days)	0.94

The intercept refers to the logarithm of the initial concentration of antioxidant compounds (ln_C0_) and the slope to the first-order constant (k). A linear trend in the data was observed in all oils.

## Data Availability

The data presented in this study are available on request from the corresponding author.

## Supplementary Materials

The following supporting information can be downloaded at: https://www.mdpi.com/article/10.3390/molecules28207045/s1, Figure S1: Chromatogram of the standard mixture FAME 37 component SUPELCO Ref CRM47885 using GC-FID; Figure S2: Chromatogram of the standard mixture PUFA No. 3 Menhaden oil Ref 47085-U using GC-FID; Figure S3: Chromatogram of plum seed oil using GC-FID and Figure S4: Chromatogram of cherry seed oil using GC-FID.

## Sample Availability

Samples of the compounds are available from the authors.
