# Supplementary material for: Valorization of Prunus Seed Oils: Fatty Acids Composition and Oxidative Stability
Source: Molecules. 2023 Oct 12;28(20):7045. doi: 10.3390/molecules28207045 (PMC10609056; doi:10.3390/molecules28207045)
Supplement: Supplementary file 1 [file molecules-28-07045-s001.zip › molecules-2638475-supplementary.pdf]

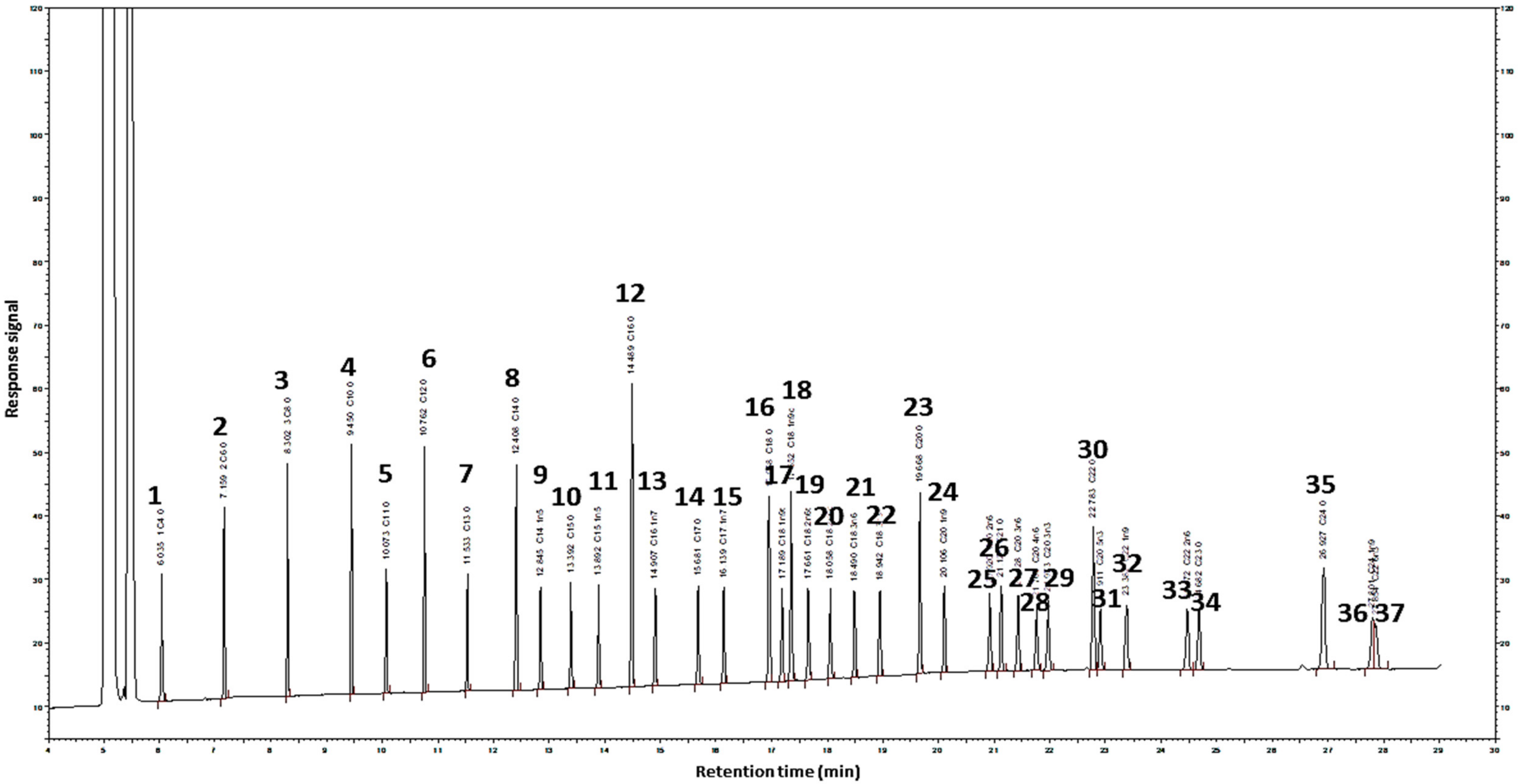

Figure S1. Chromatogram of the standard mixture FAME 37 component SUPELCO Ref CRM47885 by GC-FID. (**1**) Methyl butyrate (C4:0); (**2**) methyl hexanoate (C6:0); (**3**) methyl octanoate (C8:0); (**4**) methyl decanoate (C10:0); (**5**) methyl undecanoate (C11:0); (**6**) methyl laurate (C12:0); (**7**) methyl tridecanoate (C13:0); (**8**) methyl myristate (C14:0); (**9**) methyl myristoleate (C14:1n5); (**10**) methyl pentadecanoate (C15:0); (**11**) methyl cis-10-pentadecenoate (C15:1n5); (**12**) methyl palmitate (C16:0); (**13**) methyl palmitoleate (C16:1n7); (**14**) methyl heptadecanoate (margarate, C17:0); (**15**) cis-10-heptadecanoic acid methyl ester (C17:1n7); (**16**) methyl stearate (C18:0); (**17**) trans-9-elaidic acid methyl ester (C18:1n9t); (**18**) cis-9-oleic acid methyl ester (C18:1n9c); (**19**) methyl linolelaidate (C18:2n6t); (**20**) methyl linoleate (C18:2n6c); (**21**) methyl γ-linolenate (C18:3n6); (**22**) methyl linolenate (C18:3n3); (**23**) methyl arachidate (C20:0); (**24**) methyl cis-11-eicosenoate (C20:1n9); (**25**) cis-11,14-Eicosadienoic acid methyl ester (C20:2n6); (**26**) methyl heneicosanoate (C21:0); (**27**) cis-8,11,14-eicosatrienoic acid methyl ester (C20:3n6); (**28**) cis-5,8,11,14-eicosatetraenoic acid methyl ester (C20:4n6); (**29**) cis-11,14,17-eicosatrienoic acid methyl ester (C20:3n3); (**30**) methyl behenate (C22:0); (**31**) cis-5,8,11,14,17-Eicosapentaenoic acid methyl ester (C20:5n3); (**32**) methyl erucate (C22:1n9); (33) cis-13,16-docosadienoic acid methyl ester (C22:2n6); (**34**) methyl tricosanoate (C23:0); (**35**) methyl lignocerate (C24:0); (**36**) cis-4,7,10,13,16,19-Docosahexaenoic acid methyl ester (C22:6n3); (**37**) methyl nervonate (C24:1n9).

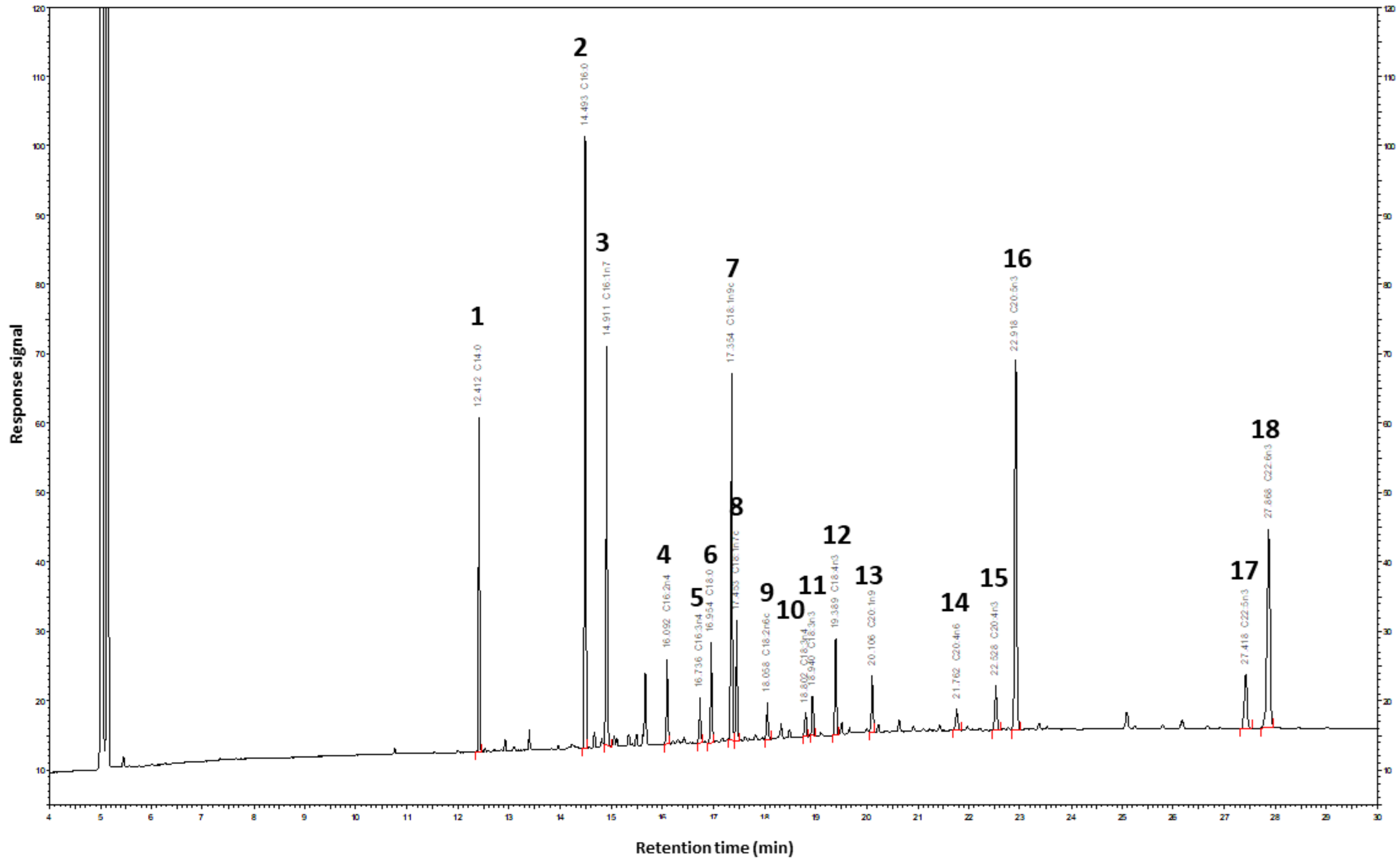

Figure S2. Chromatogram of the standard mixture PUFA Nº3 Menhaden oil Ref 47085-U by GC-FID. (1) Methyl myristate (C14:0); (**2**) methyl palmitate (C16:0); (**3**) methyl palmitoleate (C16:1n7); (**4**) cis- 9,12 hexadecadienoic acid methyl ester (C16:2n4); (**5**) cis 6,9,12-hexadecatrienoic acid methyl ester (C16:3n4); (**6**) methyl stearate (C18:0); (**7**) cis-9-oleic acid methyl ester (C18:1n9c); (**8**) cis-11 octadecenoic acid methyl ester (C18:1n7c); (**9**) methyl linoleate (C18:2n6c); (**10**) cis 9,11,14 octadecatrienoic acid methyl ester (C18:3n4); (**11**) methyl linolenate (C18:3n3); (**12**) cis-6,9,12,15 Octadecatetraenoic acid methyl ester (C18:4n3); (**13**) methyl cis-11-eicosenoate (C20:1n9); (**14**) cis-5,8,11,14 eicosatetraenoic acid methyl ester (C20:4n6); (**15**) cis-8,11,14,17 eicosatetraenoic acid methyl ester (C20:4n3); (**16**) cis-5,8,11,14,17-eicosapentaenoic acid methyl ester (C20:5n3); (**17**) cis-7,10,13,16,19 docosapentaenoic acid methyl ester (C22:5n3); (**18**) cis-4,7,10,13,16,19 docosahexaenoic acid methyl ester (C22:6n3).

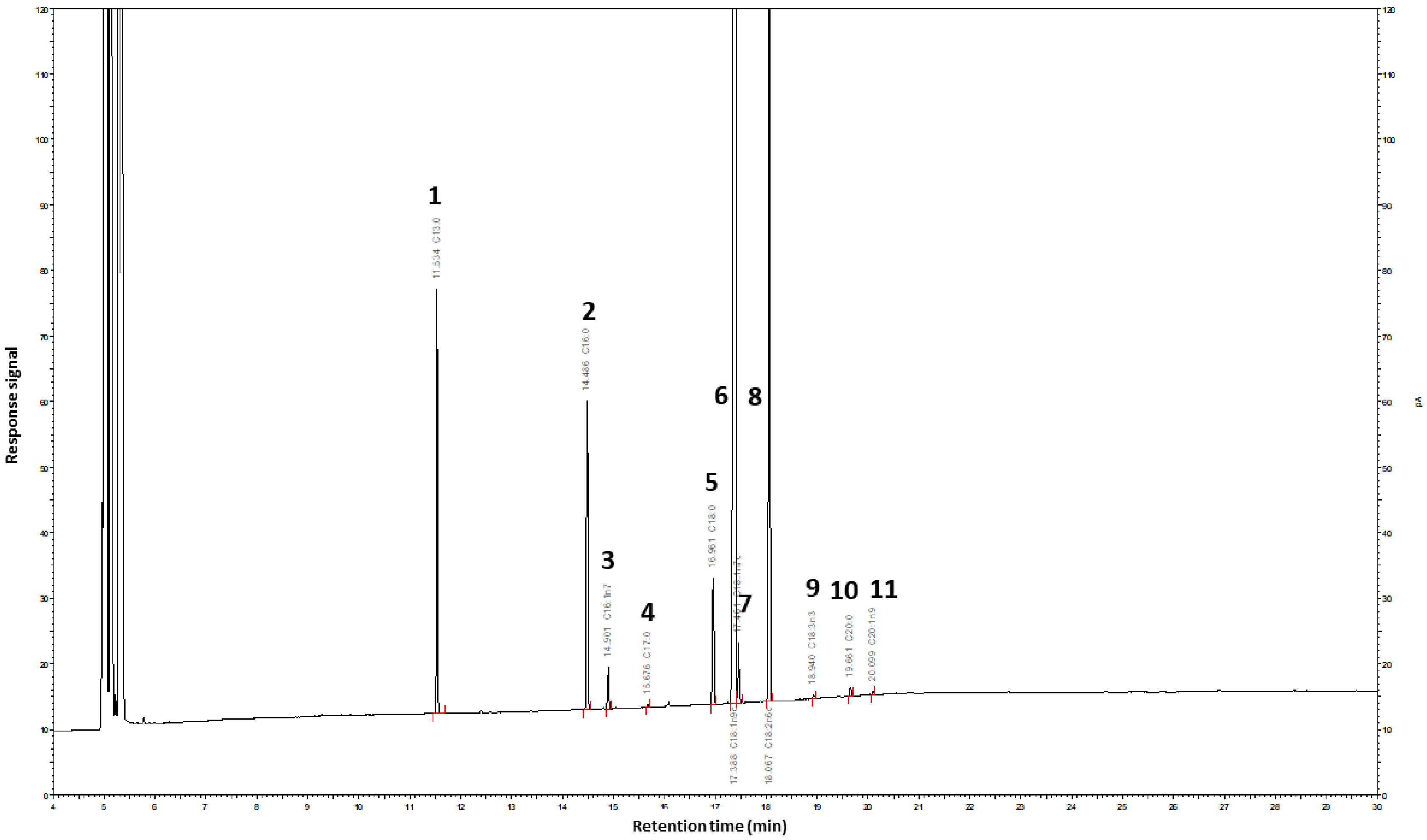

Figure S3. Chromatogram of plum seed oil by GC-FID. (**1**) methyl tridecanoate (C13:0) internal standard; (**2**) methyl palmitate (C16:0); (**3**) methyl palmitoleate (C16:1n7); (**4**) methyl heptadecanoate (margarate, C17:0); (**5**) cis-10-heptadecanoic acid methyl ester (C17:1n7); (**6**) methyl stearate (C18:0); (**7**) cis-9-oleic acid methyl ester (C18:1n9c); (**8**) methyl linoleate (C18:2n6c); (**9**) methyl linolenate (C18:3n3); (**10**) methyl arachidate (C20:0); (**11**) methyl cis-11-eicosenoate (C20:1n9).

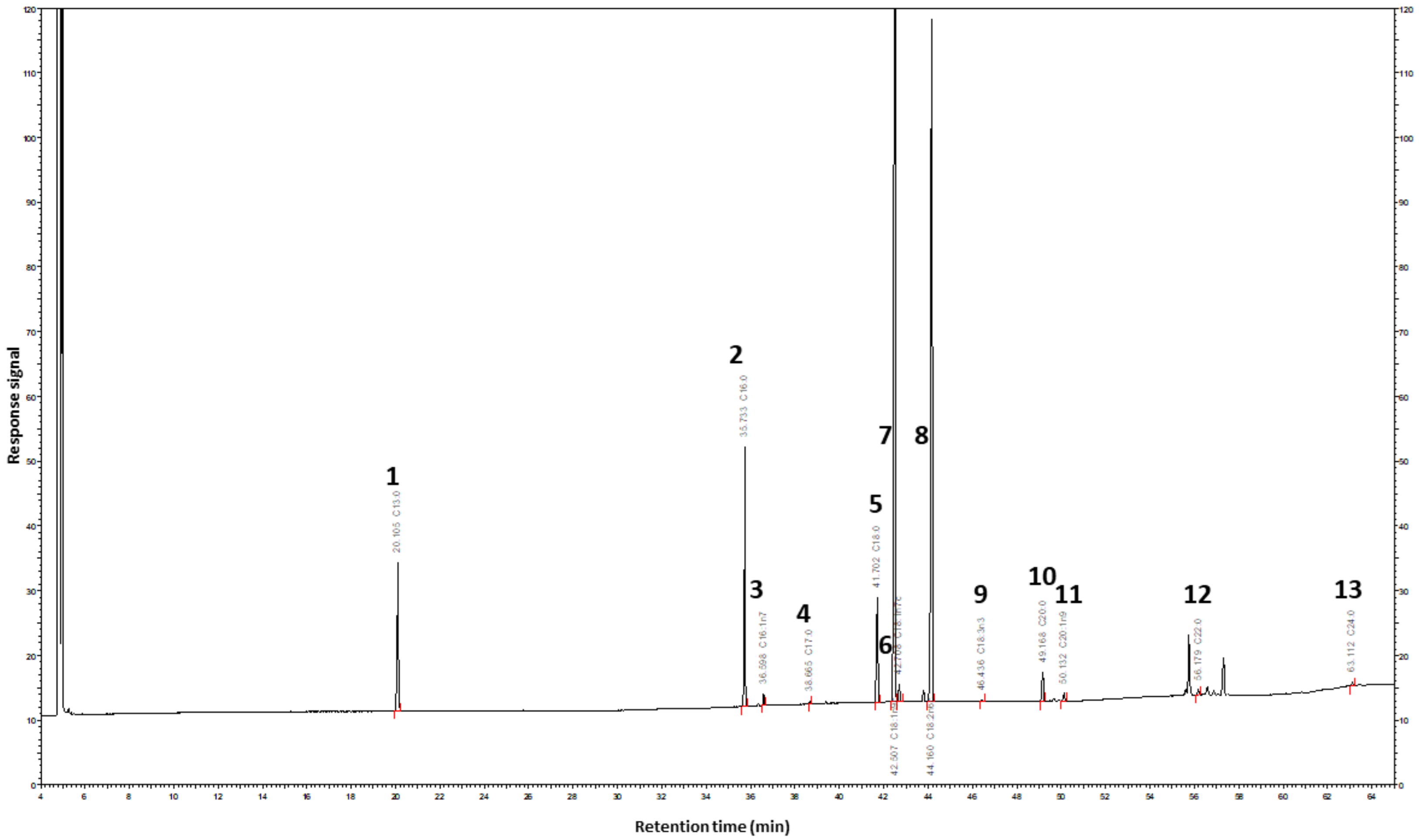

Figure S4. Chromatogram of cherry seed oil by GC-FID. (**1**) methyl tridecanoate (C13:0) internal standard; (**2**) methyl palmitate (C16:0); (**3**) methyl palmitoleate (C16:1n7); (**4**) methyl heptadecanoate (margarate, C17:0); (**5**) cis-10-heptadecanoic acid methyl ester (C17:1n7); (**6**) methyl stearate (C18:0); (**7**) cis-9-oleic acid methyl ester (C18:1n9c); (**8**) methyl linoleate (C18:2n6c); (**9**) methyl linolenate (C18:3n3); (**10**) methyl arachidate (C20:0); (**11**) methyl cis-11-eicosenoate (C20:1n9); (**12**) methyl behenate (C22:0); (**13**) methyl lignocerate (C24:0).
